# Electronic cigarettes and pregnancy: A social media content analysis☆

**DOI:** 10.1016/j.ypmed.2025.108387

**Published:** 2025-08-13

**Authors:** Grace Kong, Rachel R. Ouellette, Amanda de la Noval, Christina N. Kyriakos, Vanessa Ponte, Elise E. DeVito

**Affiliations:** Department of Psychiatry, Yale School of Medicine, New Haven, CT, United States of America

**Keywords:** E-cigarettes, Pregnancy, Social media, Qualitative study, Nicotine, Vaping, Content analysis

## Abstract

**Objective:**

Electronic cigarette (e-cigarette) use during pregnancy is a polarizing and complex public health topic. We examined social media content on e-cigarette use during pregnancy.

**Methods:**

We content analyzed 170 TikTok posts about e-cigarettes and pregnancy in 2023. We coded message valence (anti-, pro-, mixed/unclear/neutral), messenger characteristics (age, gender, pregnancy status, healthcare provider status, e-cigarette and cigarette use), geographic origin, and engagement metrics (likes, favorites, shares, downloads, comments, followers). We assessed whether engagement and message valence differed by messenger and post characteristics using Chi-Square and Kruskal-Wallis tests.

**Results:**

Posts included 55 % (*n* = 93) anti-, 32.4 % (*n* = 55) mixed/unclear/neutral, and 12.9 % (*n* = 22) pro-e-cigarette use during pregnancy messages. Messenger characteristics included 90.6 % (*n* = 144) female, 53.5 % (*n* = 85) 18 to 30 years old, 40.9 % (*n* = 65) pregnant, 7.5 % (*n* = 12) healthcare providers. Geographic origin included 66.5 % (*n* = 113) from the United States and 22.9 % (*n* = 39) from the United Kingdom. Engagement was high, with some videos receiving over 2 million “plays” and 500 thousand “likes.” Posts by healthcare providers and messengers over 45 years old had more followers (*p*s < 0.001). Posts with messengers who quit e-cigarettes, from the US, and 18–45 years old were more likely to contain anti-e-cigarette content, whereas posts from the United Kingdom were more likely to have pro-e-cigarette content (*p*s ≤ 0.001).

**Conclusions:**

A wide range of content on e-cigarette use and pregnancy was observed on TikTok. Future research is needed to understand how pregnant individuals navigate this content. Healthcare providers may be effective messengers for promoting e-cigarette cessation during pregnancy on social media.

## 1. Introduction

Electronic cigarettes (e-cigarettes) have been more commonly used than combustible cigarettes among young adults for over a decade (Arrazola et al., 2025). These young adults are at the age when pregnancy becomes more likely, making it a timely topic to explore perceptions regarding e-cigarette use during pregnancy. Although the health risks of cigarette smoking during pregnancy are well-established (United States, 2014; Morales-Prieto et al., 2021; United States, Department of Health and Human Services, 2001), the health risks of e-cigarette use during pregnancy are less researched and less communicated to the public (DeVito et al., 2021a).

Data on the health effects of e-cigarette use during pregnancy has only recently emerged and is nuanced (DeVito et al., 2021a). Human studies have shown mixed findings regarding the risks of e-cigarette use during pregnancy (DeVito et al., 2021b). Some studies have shown that either exclusive e-cigarette use (Wang et al., 2020; Regan et al., 2021; Kim and Oancea, 2020) or dual use of e-cigarettes and combustible cigarettes (Wang et al., 2020; Cardenas et al., 2019; Cardenas et al., 2020; Wang et al., 2022) increases risks of adverse perinatal outcomes (e.g., low birth weight) compared to not using e-cigarettes or combustible cigarettes. However, not all studies have found an increased risk with e-cigarette use (McDonnell et al., 2020; Cohn et al., 2023). *E*-cigarettes share some known harmful characteristics with combustible smoking (e.g., nicotine), while some harmful constituents are present in combustible smoke but not e-cigarettes (e.g., carbon monoxide) (United States, 2014). Additionally, some e-cigarettes can contain other harmful constituents that may be absent from some combustible cigarettes (e.g., flavor chemicals) (Ward et al., 2020). The currently available evidence indicates that e-cigarette use during pregnancy poses a greater risk than abstaining from all nicotine and tobacco products, including e-cigarettes and combustible cigarettes.

Examining how e-cigarette use during pregnancy is portrayed on social media platforms like TikTok may provide important insight into current perceptions of individuals who are or may become pregnant. Social media has been extensively examined to understand various trends and perceptions related to tobacco (Kwon and Park, 2020; Lee et al., 2023). Pregnant individuals frequently use the Internet, including social media, to seek pregnancy-related information and may rely on these sources instead of consulting healthcare professionals (Sayakhot and Carolan-Olah, 2016), particularly for stigmatizing topics such as e-cigarette use during pregnancy. Additionally, they may encounter e-cigarette-related topics through social media algorithms that promote content designed to increase engagement if they have searched for or viewed e-cigarette content. Therefore, examining social media content related to e-cigarettes and pregnancy can help researchers and health professionals identify perceptions that can inform clinical care and public health messaging.

Despite the importance of examining social media content regarding e-cigarette use during pregnancy, research on this topic in social media remains limited. A few studies have examined online forums to identify perceptions of e-cigarette use during pregnancy (Wigginton et al., 2017; Schilling et al., 2019) or postpartum while breastfeeding (Johnston et al., 2019). A study analyzing conversations from 13 online forums in 2015 found that discussions about the safety of e-cigarette use during pregnancy were characterized by three polarized perspectives – often involving claims not supported by empirical evidence: (1) quitting nicotine “cold turkey” is harmful to the fetus, (2) e-cigarettes are a harm reduction tool during pregnancy, and (3) e-cigarette use is harmful during pregnancy (Wigginton et al., 2017). Similarly, another study assessing 25 German online discussion forums in 2017 revealed mixed perceptions regarding perceived threats, benefits, and barriers to e-cigarette use during pregnancy, which were defined by themes of relative harmfulness compared to combustible cigarettes, concerns about health risks, social stigma, and lack of knowledge (Schilling et al., 2019). To our knowledge, no studies have specifically examined content on e-cigarette use during pregnancy on popular social media platforms commonly used by young adults, such as TikTok.

We aimed to understand how e-cigarette use during pregnancy is portrayed on TikTok (i.e., message valence such as pro- or anti-e-cigarette use during pregnancy), messenger characteristics (e.g., gender, age), engagement with the posts (e.g., number of plays and likes), and post characteristics (e.g., geographic region), and whether engagement varied based on these factors. Additionally, we examined whether the message valence differed by messenger and post characteristics.

## 2. Methods

### 2.1. Data obtainment

This observational study of publicly available data was deemed exempt as human subjects research by the Yale Institutional Review Board (HIC# 2000035051). To identify e-cigarette and pregnancy content, we searched for combinations of six terms (e.g., “vaping” + “pregnant”; Fig. S1) using a TikTok video scraper, Apify (Apify, 2024), on September 2023, in the United States (US). For each keyword combination, we obtained metadata, including the number of plays, likes, shares, favorites, downloads, comments, followers, and geographic origin of the post for 100 unique posts (Total *N* = 600). We downloaded the videos using Tokdownload.com. Of the 600 posts, we excluded 430 because they did not have e-cigarette and/or pregnancy content, were unavailable, or did not meet other inclusion criteria (e.g., language other than English and Spanish) (Fig. S1).

### 2.2. Procedures

We developed a codebook through a combination of deductive and inductive approaches. Deductive codes were informed by previous codebooks used by the study team to characterize social media content, including the perceived demographics (i.e., gender, age) of messengers (person[s] featured on the videos) (Ouellette et al., 2023). The study team identified additional deductive codes, such as pregnancy status, e-cigarette and cigarette use, and relevant professions such as a healthcare provider. Finally, inductive codes were identified to capture the message valence (e.g., pro- or anti-e-cigarette use) related to e-cigarette use during pregnancy reflected in the TikTok posts. Inductive codes were identified through an iterative review of posts by four team members, who independently reviewed 10–30 posts each and then discussed codebook revisions as a group until saturation was met (i.e., no new codes were identified). Coding was initially conducted in Excel, with final codes entered into SPSS for data analysis (see statistical analysis). We determined interrater reliability by double coding a random selection of 11.7 % (*n* = 20) of videos.

### 2.3. Message, messenger, and post characteristics

#### 2.3.1. Message valence

We coded message valence regarding e-cigarette use during pregnancy as pro-e-cigarette use, anti-e-cigarette use, mixed (containing multiple valences such as pro-, anti-, and neutral content), unclear (unable to determine valence), or neutral (neither pro- nor anti-e-cigarette use). We combined mixed, unclear, and neutral content.

#### 2.3.2. Messenger characteristics

For every post featuring humans, we assessed perceived gender (female, male, “uncertain”), perceived age (≤17 years, 18 to 30 years, 31 to 45 years, > 45 years [age where pregnancy is unlikely]), e-cigarette use (i.e., unknown, quit e-cigarette use, currently use e-cigarettes, never used e-cigarettes, trying to cut down or quit e-cigarette use), cigarette smoking (same categories as e-cigarette use), pregnancy status (pregnant, unknown, previously pregnant, planning to become pregnant, stated not pregnant), and healthcare provider status (yes, no). Most posts featured a single person; in rare cases where more than one person was shown, we coded for the focal person - the individual who spoke the most or appeared most prominently in the posts. Pregnancy status, healthcare provider, e-cigarette use, and cigarette smoking were determined based on the contextual content of the posts, including explicit statements or visible cues (e.g., smoking, using e-cigarettes, wearing scrubs).

#### 2.3.3. Engagement metrics

The engagement data were obtained from metadata, which included the number of times each video was played (not counting replays), liked, shared, favorited, downloaded, and commented on, and the number of followers the poster had.

#### 2.3.4. Post characteristics

The geographic origin of the post was obtained from metadata.

### 2.4. Statistical analysis

We used Kruskal-Wallis tests to assess the association between engagement metrics and message valence, demographic characteristics, geographic origin of the posts, pregnancy status, healthcare provider status, e-cigarette use status, and cigarette smoking status. We selected Kruskal-Wallis tests because of the positively skewed engagement metrics data. We applied a Bonferroni correction to account for multiple comparisons and set a significance *p*-value of ≤0.007 (0.05 divided by the seven engagement variables). Cohen’s Kappa (κ) was used to assess inter-rater reliability.

We also conducted chi-square tests to assess whether message valence differed by demographic characteristics, geographic origin of the posts, pregnancy status, healthcare provider status, e-cigarette use status, and cigarette smoking status. We combined any categories with less than 5.0 % of the sample for these analyses. For instance, we grouped countries that appeared less than 5.0 % into an “other” countries category. The only exception was the age ≤ 17 age category (3.1 %). We retained this age as its own category, as all other age categories exceeded 5.0 %, and meaningful aggregation was not feasible. Descriptive statistics, bivariate analyses, and reliability analysis were calculated using SPSS Version 29 (Wigginton and Lafrance, 2016).

## 3. Results

### 3.1. Message valence

The message valence was 54.7 % (*n* = 93) anti-e-cigarette use during pregnancy, 12.9 % (*n* = 22) pro-e-cigarette use during pregnancy, and 32.4 % (*n* = 55) “mixed, unclear, or neutral.” The subthemes for each valence are described below, listed from most common to least, with detailed examples in Table 1. Interrater reliability ranged from substantial to near perfect (κs 0.72 to 1.0, *p*s ≤ 0.001) across codes.

#### 3.1.1. Anti-e-cigarette use

Themes included 1) testimonials (i.e., self-report of personal experience) about quitting e-cigarette use while pregnant. This content was mostly related to quitting e-cigarettes because of concerns about e-cigarette use on the baby’s and maternal health. Other testimonials included methods of quitting and comments expressing cutting down gradually because of concerns that quitting cold turkey might harm the baby more than nicotine could; 2) shaming or criticizing people who use e-cigarettes while pregnant and referring to them as “gross,” “disgusting,” “trashy,” and “selfish;” 3) urging people not to use e-cigarettes during pregnancy because of negative health effects; 4) urging people not to use e-cigarettes because they negatively impact fertility; and 5) less common themes included rejecting other people’s posts that portray e-cigarette use during pregnancy as acceptable, advising others not to use e-cigarettes while breastfeeding or serving as a surrogate, and quitting during pregnancy but anticipating resuming e-cigarette use after childbirth.

#### 3.1.2. Pro-e-cigarette use

Themes included 1) testimonials of using e-cigarettes during pregnancy (some of which included showing a healthy baby while stating that the baby was unaffected by e-cigarette use during pregnancy); 2) testimonials of switching from cigarette smoking to e-cigarette use, often framing e-cigarettes as a safer option than combustible cigarettes and other substances used during pregnancy; 3) claims of e-cigarette use during pregnancy as being safe, including hearsay from mothers and doctors saying that e-cigarette use during pregnancy was safe; and 4) other less common themes included using e-cigarettes for birth control, since e-cigarette use can negatively impact fertility.

#### 3.1.3. Mixed, unclear, or neutral

Themes included 1) assertions that e-cigarette use during pregnancy is a personal choice and should not be subject to external judgment; 2) content that did not explicitly express an opinion on e-cigarette use during pregnancy or mentioned the topic in unrelated ways; and 3) other less common themes included jokes about using or quitting e-cigarette use during pregnancy, celebrity gossip about using e-cigarettes in the presence of pregnant people, and a mix of anti- and pro-valence.

### 3.2. Messenger characteristics

Table 2 presents messenger characteristics. 93.5 % (*n* = 159) of the posts included humans. Of these, 90.6 % (*n* = 144) of messengers were perceived as female, and the most common perceived age was 18–30 years old (53.5 %; *n* = 85) followed by 31–45 years old (30.2 %; *n* = 48). A total of 40.9 % (*n* = 65) were pregnant, and 7.5 % (*n* = 12) were healthcare providers (e.g., midwife, obstetrician-gynecologist, dental hygienist). The top e-cigarette use status was “unknown” (44.7 %; *n* = 76), followed by 27.1 % (*n* = 46) who have quit e-cigarettes, and 22.4 % (*n* = 38) currently using e-cigarettes. The top cigarette smoking status was “unknown” (93.5 %; *n* = 159), followed by 3.5 % (*n* = 6) who have quit smoking, and 2.4 % (*n* = 4) who were currently smoking.

### 3.3. Geographic origin of the posts

Posts originated from the US 66.5 % (*n* = 113), United Kingdom (UK) 22.9 % (*n* = 39), and “other” countries 10.6 % (*n* = 18: Canada [n = 6], New Zealand [n = 4], Australia [n = 3], Philippines [n = 3], Mexico [*n* = 2]; Table 2).

### 3.4. Engagement metrics

The median number of plays was 12,950 (IQR = 59,986), likes 443.5 (IQR = 3235), shares 8.0 (IQR = 50), favorites 15.5 (IQR = 115), comments 21.5 (IQR =103), downloads 0 (IQR =5), and followers 6805 (IQR = 46,138). Notably, the maximum number of followers was 2,981,372, plays was 2,745,730, and likes was 536,562 (Table 3).

### 3.5. Analysis by engagement metrics and study variables

The number of followers was significantly higher in posts where messengers were healthcare providers (Med = 116,167, IQR = 1,273,926) versus non-healthcare providers (Med = 6795, IQR = 43,432), H(1) = 10.82, *p <* .001). The number of followers also differed by messengers’ perceived age (H(4) = 26.36, p < .001), with >45 years (Med = 93,319, IQR = 99,989) having the greatest number of followers followed by 31–45 years (Med = 18,297, IQR = 101,326), ≤17 years (Med = 5314, IQR =62,999), 18–30 years (Med = 5030, IQR =25,782), and unknown or not applicable age (Med = 361, IQR = 1466). No significant associations were observed between other engagement metrics and other study variables.

### 3.6. Analysis by message valence and study variables

Age (χ^2^ [8, 159] = 37.57, *p <* .001), e-cigarette use status (χ^2^ [6, 159] = 88.32, *p <* .001, and geographic region (χ^2^ [4, *N* = 170] = 18.01, *p* = .001) were significantly associated with message valence (Table 4). Specifically, posts from adults aged 18–30 years and 31–45 years had the most anti-e-cigarette content, and “unknown” age had the most pro-e-cigarette content. Individuals who quit e-cigarettes were more likely to share anti-e-cigarette use content than pro or mixed/unclear/neutral content. Additionally, posts from the US were more likely to have anti-e-cigarette content. In contrast, posts from the UK were more likely to have pro-e-cigarette content compared to posts from other regions. There were no other significant differences between message valence and other study variables (Table S1).

## 4. Discussion

A content analysis of TikTok posts on e-cigarettes and pregnancy showed varied and even conflicting content regarding e-cigarette use during pregnancy: 55 % of the posts contained anti-e-cigarette use content, 32 % featured mixed, unclear, or neutral content, and 13 % contained pro-e-cigarette content. Messengers were female adults of ages where childbirth is likely (53.5 % 18–30 years) and people currently pregnant or previously pregnant (62.4 %). The most viewed videos received over two million views and liked more than 500 thousand times.

The predominant themes within anti-e-cigarette use message valence included testimonials from individuals sharing their experiences quitting e-cigarettes during pregnancy and urging others not to use e-cigarettes while pregnant due to health risks. Anti-e-cigarette content also included individuals shaming and criticizing pregnant people who use e-cigarettes. Pro-e-cigarette content included testimonials from pregnant people or previously pregnant people regarding using e-cigarettes during pregnancy, switching from cigarette to e-cigarette use, and claims that e-cigarette use is safe during pregnancy. Mixed, unclear, and neutral content included testimonials from pregnant people expressing excitement to resume e-cigarette use after childbirth, as well as individuals reporting being addicted to and guilty about using e-cigarettes during pregnancy but continuing to do so. The conflicting valence of e-cigarette use during pregnancy has been identified in other media forms, such as newspaper articles and online forum discussions (Wigginton et al., 2017; Schilling et al., 2019; Moyse and Hunter, 2021). These prior studies have also shown that anti-e-cigarette content highlighted potential health risks, while pro-e-cigarette content emphasized harm reduction benefits, framing e-cigarette use as the “lesser of two evils” compared to cigarettes and other substances.

Concerningly, some posts contained misinformation. One claim, which emerged repeatedly, was that abruptly quitting e-cigarette use (or combustible cigarettes) could harm the baby by inducing stress more than continued use would. However, scientific evidence clearly shows that quitting such products is beneficial during pregnancy, and there is no evidence that temporary withdrawal symptoms, such as stress and anxiety, cause more harm than continued use (Xie et al., 2023). However, this myth persists, as other research has similarly observed, in online forums (Wigginton et al., 2017). Other misinformation was that e-cigarettes could be effectively used for birth control. While e-cigarettes, and combustible cigarettes, can negatively impact fertility (Bhardwaj et al., 2025), they are not developed or tested for this purpose. These findings demonstrate the need for effective strategies to combat misinformation and convey accurate health information on social media.

The e-cigarette use status of messengers was mixed, with 27 % indicating they had quit e-cigarette use and 22 % indicating they currently used e-cigarettes. Disclosure of cigarette smoking was rare; the majority (94 %) did not disclose their cigarette smoking status, and very few reported quitting smoking (4 %) or currently smoking (2 %). The fact that few of the posts mentioned cigarette smoking is noteworthy because prior studies indicated that people who use e-cigarettes during pregnancy also have a history of cigarette smoking or engage in dual use (Kim and Oancea, 2020; Wang et al., 2022; McDonnell et al., 2020; Cohn et al., 2023). This pattern has often been interpreted as evidence that e-cigarette use during pregnancy may reflect attempts to switch from cigarettes to e-cigarettes or to dual use of e-cigarettes to cut down or quit smoking cigarettes. Several factors may explain our finding. One possibility is that these social media data reflect the behaviors and perceptions of younger cohorts who may be more likely to use e-cigarettes than prior generations. Indeed, data indicate that young adult females are less likely to smoke cigarettes and more likely to use e-cigarettes than older adults (Kramarow and Elgaddal, 2023). This suggests that unique considerations regarding health messages surrounding e-cigarette use during pregnancy are warranted. Another possibility is that public health messaging regarding the negative health effects of cigarette smoking is well-communicated to the public, and there is stigma associated with cigarette smoking during pregnancy (Estey et al., 2024). As a result, pregnant individuals may be less inclined to disclose their cigarette smoking status on social media, as has been observed in other studies (Wigginton and Lafrance, 2016).

Our findings also underscore the challenges some pregnant individuals encounter when attempting to quit e-cigarettes. They expressed conflicted feelings about their addiction to e-cigarettes and felt guilty about using them, yet they were unable to stop. These findings highlight the need for effective interventions that provide support to help pregnant individuals quit e-cigarette use while addressing the potential feelings of guilt and shame associated with e-cigarette use. Some pregnant individuals who have quit expressed excitement about resuming e-cigarette use after giving birth. While studies on the relapse of e-cigarettes are lacking, studies that examined cigarette smoking showed that the postpartum cigarette smoking relapse rate is high and interventions to prevent postpartum smoking have been limited and generally ineffective (Chamberlain et al., 2017; Levine et al., 2016). Focusing efforts to motivate pregnant individuals to stay abstinent from e-cigarettes and cigarette smoking after giving birth is a crucial public health goal.

Social media can facilitate the cross-border spread of health information across countries with varying guidelines on the use of e-cigarettes during pregnancy. Although we searched TikTok from within the US, we found that a third of the posts originated from outside the country, most notably from the UK. TikTok posts from the UK were more likely than those from the US or other countries to be pro-e-cigarette. The greater number of pro-e-cigarette posts from the UK may reflect this country’s public health stance that expresses more support for the use of e-cigarettes as a harm reduction tool and has approved one for use during pregnancy to encourage switching among pregnant individuals who smoke (van der Eijk et al., 2017). In contrast, the World Health Organization and US-based organizations warn women against using e-cigarettes while pregnant (Center for Disease Control, 2025; World Health Organization, 2025). For pregnant individuals, these conflicting guidelines from within their own country as well as from other countries may create confusion.

Given the presence of content related to e-cigarette use during pregnancy on social media, future studies should examine factors such as messenger characteristics and other components that make health messaging more effective on this platform. For example, although healthcare providers were minimally present in our data, they had the highest number of followers. These healthcare providers were obstetrician-gynecologists, midwives, and dental hygienists who provided a wide range of health information, including e-cigarette and combustible tobacco use during pregnancy. This finding suggests that healthcare providers who have a large following on social media may be leveraged to provide accurate and convincing health information and motivate people to quit e-cigarette use, as well as combustible tobacco use, during pregnancy.

Limitations should be considered when interpreting the study findings. Social media may present polarizing or extreme views (Prasetya and Murata, 2020). Therefore, other methods, such as surveys, are necessary to ensure that views and perceptions observed in social media are representative. Despite this limitation, social media can offer valuable insight into people’s perceptions of emerging, less-studied topics that may be stigmatizing. Another limitation is that we did not directly assess the messengers’ demographics and nicotine/tobacco use history; instead, we inferred these characteristics based on appearance and other information they provided in the post. This also resulted in a substantial number of cases categorized as “unknown.” However, this type of coding is commonly used in digital media research when such information is unavailable (Ouellette et al., 2023). Additionally, we did not record the gender and age of every individual in select cases where multiple people were included; instead, we identified the gender and age of the person most prominently featured.

In summary, we identified a range of diverse and contradictory content regarding e-cigarette use during pregnancy on TikTok. Given that social media is a powerful tool for communicating health information, future research should evaluate how pregnant individuals interact with and navigate this diverse information. Healthcare providers should be cognizant of this varied content on social media to counteract harmful misinformation that may serve as effective messengers for promoting e-cigarette cessation during pregnancy. Lastly, while we investigated content related to e-cigarette use and pregnancy, we also observed many related topics concerning pregnancy, such as fertility, surrogacy, and breastfeeding. Future studies should further explore e-cigarette use across these various stages before, during, and after pregnancy.

## Supplementary Material

MMC1

## Figures and Tables

**Table 1:** Descriptive statistics of main themes and description of subthemes of message valence from 170 TikTok posts with e-cigarette use and pregnancy content collected in September 2023.

Main themes	% (n)	Subthemes

1. Anti-e-cigarette use during pregnancy	54.7 (93)	Testimonials about quitting e-cigarette use during pregnancy (e.g., reasons for quitting such as health harm to the baby, methods of quitting such as gradually cutting down or getting rid of their e-cigarettes) Shaming and criticizing people who use e-cigarettes during pregnancy Urging people not to use e-cigarettes during pregnancy because of negative health effects Urging people not to use e-cigarettes because of negative effects on fertility “Other”: Discussing requirements to be a surrogate (e.g., cannot smoke or vape) or during breastfeeding; reacting to others’ posts about e-cigarette use being safe
2. Pro-e-cigarette use during pregnancy	12.9 (22)	Testimonials of using e-cigarette during pregnancy (e.g., showing a healthy baby while stating that the baby was unaffected by e-cigarette use during pregnancy, disclosing being addicted to nicotine and an inability to quit, expressing concerns that quitting cold Turkey and experiencing withdrawal could harm the baby or cause miscarriages) Testimonials of switching from cigarette smoking to vaping Discussion of e-cigarette use during pregnancy as safe “Other”: e-cigarette use as a source of birth control
3. Mixed/unclear/neutral valence regarding e-cigarette use during pregnancy	32.4 (55)	Assertions that e-cigarette use during pregnancy is a personal choice and not subject to external judgment No explicit opinions, e-cigarettes and pregnancy mentioned in unrelated ways Testimonials from pregnant people expressing anticipation of resuming e-cigarette use after childbirth “Other”: Jokes/sarcasm about using or quitting e-cigarette use during pregnancy, celebrity gossip about using e-cigarettes in the presence of pregnant people, testimonials about being addicted to e-cigarettes and trying to convince themselves to quit e-cigarette use during pregnancy, and mixed valence (e.g., testimonials of feeling guilty because secretly used e-cigarettes while pregnant; being pro-quitting e-cigarettes but anti-quitting cold Turkey).

**Table 2:** Descriptive statistics of messenger characteristics and geographic origin of 170 TikTok posts with e-cigarettes and pregnancy content collected in September 2023.

Messenger characteristics	% (n)^1^

Perceived gender	
Female	90.6 (144)
Male	8.8 (14)
Cannot be determined	0.6 (1)
Perceived age	
≤17 years old	3.1 (5)
18–30 years old	53.5 (85)
31–45 years old	30.2 (48)
> 45 years old	7.5 (12)
Cannot be determined	5.7 (9)
Pregnancy status	
Pregnant	40.9 (65)
Unknown	34.6 (55)
Previously pregnant	21.4 (34)
Planning to become pregnant	1.3 (2)
Stated not pregnant	1.9 (3)
Healthcare provider	
Yes	7.5 (12)
No	94.3 (158)
*E*-cigarette use status	
Unknown	44.7 (76)
Quit e-cigarette use	27.1 (46)
Currently use e-cigarettes	22.4 (38)
Never used e-cigarettes	3.5 (6)
Trying to cut down or quit e-cigarettes	2.4 (4)
Cigarette smoking status	
Unknown	93.5 (159)
Quit smoking	3.5 (6)
Currently smoking	2.4 (4)
Never smoked	0.6 (1)
Trying to cut down or quit smoking	0
Geographic origin	
United States	66.5 (113)
United Kingdom	22.9 (39)
Other^2^	10.6 (18)

1 The percentages for messenger characteristics were derived from 159 posts that featured humans.

2 _“_Other” category included: Canada (3.5 %, *n* = 6), New Zealand (2.4 %, *n* = 4), Australia (1.7 %, *n* = 3), Philippines (1.7 %, n = 3), Mexico (1.2 %, *n* = 2).

**Table 3:** Descriptive statistics of engagement metrics of 170 TikTok posts with e-cigarette and pregnancy content collected in September 2023.

	All posts (N = 170)		
	Med (IQR)	Min	Max

Plays	12,950 (59,986)	327	2,745,730
Likes	443.5 (3235)	1	536,562
Shares	8.0 (50)	0	37,423
Favorites	15.5 (115)	0	24,109
Comments	21.50 (103)	0	10,580
Downloads	0 (5)	0	1398
Followers	6805 (46,138)	20	2,981,372

**Note:** Med = Median, IQR = interquartile range, Min = minimum, Max = maximum.

**Table 4:** Chi-square analyses and descriptive statistics examining differences in message valence by age and e-cigarette use status of messengers and geographic origin of 170 TikTok posts with e-cigarette and pregnancy content collected in September 2023.

	Anti-e-cigarette	Pro-e-cigarette	Mixed, unclear, neutral	P value^1^
	% (n)			

Age				
≤17 years	0.0 (0)	0.0 (0)	10.9 (5)	<0.001
18–30 years	57.1 (52)	50 (11)	47.8 (22)	
31–45 years	35.2 (32)	18.2 (4)	26.1 (12)	
>45 years	6.6 (6)	4.5 (1)	10.9 (5)	
Unknown	1.1 (1)	27.3 (6)	4.3 (2)	
E-cigarette use status**				
Unknown	42.9 (39)	18.2 (4)	60.9 (28)	<0.001
“Other” use status	4.4 (4)	0 (0)	13 (6)	
Quit already	47.3 (43)	0 (0)	4.3 (2)	
Geographic origin				
US	77.4 (72)	40.9 (9)	58.2 (32)	0.001
UK	11.8 (11)	50.0 (11)	30.9 (17)	
“Other”	10.8 (10)	9.1 (2)	10.9 (6)	

**Note:** We grouped countries that appeared less than 5 % (Canada [3.5 %, n = 6], New Zealand [2.4 %, n = 4], Australia [1.7 %, n = 3], Philippines [1.7 %, n = 3], Mexico [1.2 %, n = 2]) into an “other” countries category.

1 P-values are derived from chi-square analyses.

## Data Availability

Data will be made available on request.

## Appendix A. Supplementary data

Supplementary data to this article can be found online at https://doi.org/10.1016/j.ypmed.2025.108387.
