# Supplementary material for: Electronic cigarettes and pregnancy: A social media content analysis
Source: Prev Med. Author manuscript; Available in PMC 2026 Jan 20. (PMC12818053; doi:10.1016/j.ypmed.2025.108387)
Supplement: MMC1 [file NIHMS2107749-supplement-MMC1.docx]

**Supplementary Figure 1.** Flow diagram illustrating the number of TikTok posts included and excluded in the study, along with reasons for exclusion; data collected in September 2023.

100 Unique TikTok posts for each of the six search term combinations: “vaping” + “pregnant,” “vaping” + “pregnancy,” “vape” + “pregnant,” “vape” + “pregnancy,” “e-cigarette” + “pregnant,” “e-cigarette” + “pregnancy”

Total N=600

430 (71.7%) Excluded posts

- 280 (46.5%) No e-cigarettes, yes pregnancy
- 53 (8.8%) No e-cigarettes, no pregnancy
- 41 (6.8%) Yes e-cigarettes, no pregnancy
- 32 (5.3%) Not available
- 24 (4.0%) Other

170 (28.3%) Eligible Posts

**Supplementary Table 1.** Chi-square analyses and descriptive statistics examining differences in message valence by messenger type among 159 TikTok posts with e-cigarette and pregnancy content, collected in September 2023

|  | **Anti-e-cigarette**  **(n=91)** | **Pro-e-cigarette**  **(n=22)** | **Mixed, Unclear, Neutral (n=46)** |
| --- | --- | --- | --- |
| **Gender** |  |  |  |
| Female | 94.5% (n=86) | 90.9% (n=20) | 82.6% (n=38) |
| Male | 5.5% (n=5) | 9.1% (n=2) | 15.2% (n=7) |
| Cannot be determined | 0% (n=0) | 0% (n=0) | 2.2% (n=1) |
| **Pregnancy Status** |  |  |  |
| Unknown | 26.4% (n=24) | 13.6% (n=3) | 32.6% (n=15) |
| Pregnant | 42.9% (n=39) | 68.2% (n=15) | 23.9% (n=11) |
| Previously pregnant | 23.1% (n=21) | 13.6% (n=3) | 21.7% (n=10) |
| Planning to become  pregnant | 2.2% (n=2) | 0% (n=0) | 0% (n=0) |
| Stated not pregnant | 0% (n=0) | 0% (n=0) | 6.5% (n=3) |
| Not applicable | 5.5% (n=5) | 4.5% (n=1) | 15.2% (n=7) |
| **Healthcare provider** |  |  |  |
| No | 89% (n=81) | 100% (n=22) | 95.7% (n=44) |
| Yes | 11% (n=10) | 0% (n=0) | 4.3% (n=2) |
| **Cigarette smoking status** |  |  |  |
| Unknown | 95.6% (n=87) | 90.9% (n=20) | 93.5% (n=43) |
| Never smoked | 1.1% (n=1) | 0% (n=0) | 0% (n=0) |
| Quit smoking | 3.3% (n=3) | 9.1% (n=2) | 2.2% (n=1) |
| Currently smoking | 0% (n=0) | 0% (n=0) | 4.3% (n=2) |

**Note:** Chi-square analyses did not yield any significant association between message valence and gender, pregnancy status, health care provider, or cigarette smoking status. Please note that any categories representing less than 5% of the sample were combined for bivariate analyses. Only the posts where humans appeared were included (n=159).
